# Mammalian spermatozoa and cumulus cells bind to a 3D model generated by recombinant zona pellucida protein-coated beads

**DOI:** 10.1038/s41598-019-54501-7

**Published:** 2019-11-29

**Authors:** Julieta Gabriela Hamze, Analuce Canha-Gouveia, Blanca Algarra, María José Gómez-Torres, María Concepción Olivares, Raquel Romar, María Jiménez-Movilla

**Affiliations:** 1 Department of Cell Biology and Histology, School of Medicine, University of Murcia, Campus Mare Nostrum and IMIB-Arrixaca, Murcia, Spain; 2 Department of Physiology, Faculty of Veterinary, University of Murcia, Campus Mare Nostrum and IMIB-Arrixaca, Murcia, Spain; 3 Department of Biotechnology, Chair Human Fertility, University of Alicante, Alicante, Spain; 4 Department of Biochemistry and Molecular Biology B and Immunology School of Medicine, University of Murcia, Campus Mare Nostrum and IMIB-Arrixaca, Murcia, Spain

**Keywords:** Biological models, Medical research

## Abstract

The egg is a spherical cell encapsulated by the zona pellucida (ZP) which forms a filamentous matrix composed of several glycoproteins that mediate gamete recognition at fertilization. Studies on molecular mechanisms of sperm-egg binding are limited in many mammalian species by the scarcity of eggs, by ethical concerns in harvesting eggs, and by the high cost of producing genetically modified animals. To address these limitations, we have reproduced a three-dimensional (3D) model mimicking the oocyte’s shape, by means of magnetic sepharose beads coated with recombinant ZP glycoproteins (B_ZP_) and cumulus cells. Three preparations composed of either ZP2 (C and N-termini; B_ZP2_), ZP3 (B_ZP3_) or ZP4 (B_ZP4_) were obtained and characterized by protein SDS-PAGE, immunoblot and imaging with confocal and electron microscopy. The functionality of the model was validated by adhesion of cumulus cells, the ability of the glycoprotein-beads to support spermatozoa binding and induce acrosome exocytosis. Thus, our findings document that ZP-beads provide a novel 3D tool to investigate the role of specific proteins on egg-sperm interactions becoming a relevant tool as a diagnostic predictor of mammalian sperm function once transferred to the industry.

## Introduction

Sexual reproduction is used by different organisms and involves the fusion of two highly specialized haploid cells, the egg and the spermatozoon. To ensure fertilization, sperm first must cross the cumulus cells surrounding the egg and then, contact, recognize and penetrate the extracellular egg coat (zona pellucida, ZP) to fuse with the plasma membrane. Initial sperm-egg recognition on the surface of the ZP is an essential step for fertilization and biochemical removal of the ZP matrix or its absence in gene-edited mice causes infertility^1–3^. Despite the crucial importance of fertilization, our knowledge about the molecular basis of this interaction is limited in humans and mice^4,5^ and basically non-existent in other mammalian species.

A major challenge to obtaining conclusive results on specific molecules involved in sperm-ZP binding is due to limitations on obtaining and manipulating gametes. For example, in some mammalian species it is not feasible to get capacitated sperm and matured oocytes due to ethical concerns (i.e., humans) or restricted number of gametes (i.e., endangered species). There are also technical challenges in assaying and evaluating sperm-egg interactions which is a dynamic process involving multiple macromolecules. In recent years, three-dimensional (3D) systems have become a valuable tool to study and improve our understanding of different stages of reproduction^6,7^. A 3D system recreating the spherical shape oocytes and the biochemical characteristics of the ZP would be a powerful means to investigate unsolved issues related to *in vitro* fertilization^8^.

The mammalian ZP is composed of either three or four glycoproteins designated as ZP1, ZP2, ZP3 and ZP4. In mice, the zona matrix contains ZP1, ZP2 and ZP3. ZP4 is encoded by a pseudogene that does not express the cognate protein^9^. Although the zona matrices in pig^10^, cow^11^ and dog^9^ oocytes also are made up of 3 ZP proteins, these ZP matrices lack ZP1 (rather than ZP4) which is a pseudogene in these species^9^. The ZP matrices of other mammals including rat^12^, hamster^13^, bonnet monkey^14^ and human^15^ contain all four glycoproteins. Depending on the mammalian species, each ZP glycoprotein has been proposed as a ligand for sperm^16–20^. For example, in mouse and human, sperm bind to the N-terminus of ZP2^20,21^ whereas in pigs and cows, ZP3 and/or ZP4 has been implicated in sperm-egg interaction^18,19^. This suggests that the role of individual ZP glycoproteins during fertilization may differ among mammals and needs to be investigated independently rather than extrapolating the findings of one species to another. Such investigations would be facilitated by *in vitro* model systems incorporating order-specific recombinant zona glycoproteins for validation of sperm-zona interactions in different mammals.

The contribution of individual ZP proteins to gamete recognition has been studied biochemically based on blocking potential sperm-ZP interactions with solubilized ZP^22–24^, purified native ZP proteins^25^ and recombinant ZP proteins^26–28^. In addition, antibodies directed against specific epitopes have been used to evaluate the candidacy of particular zona proteins in gamete recognition^29^. In recent years, the ease of establishing gene-edited mice has opened the possibility of studying the role of ZP glycoproteins *in vivo* which has provided new insights into mouse and human fertilization^20^. Based on a ZP2-cleavage model of gamete recognition, it has been shown that the N-terminus of ZP2 attached agarose beads can decoy sperm and prevent fertilization *in vitro* and *in vivo*^21^. These same peptide beads can be used to select human sperm that appear to have enhanced ability to bind and penetrate a zona matrix in which human ZP2 replaces endogenous mouse ZP2 and may prove useful in assisted reproductive technologies^30^. Although the use of double and triple transgenic animals can potentially serve as models, the establishment of such animals is time-intensive, expensive and mostly restricted to mice which may not provide the best model for other mammals. Thus, there is considerable interest in developing new strategies to decode sperm-egg recognition^31–33^ which will advance our understanding of *in vitro* fertilization and improve assisted human reproduction and livestock production. In this study, a new *in vitro* model is proposed and validated. The model is based on magnetic sepharose beads (B) coated with single recombinant ZP glycoproteins (B_ZP_) that mimic the 3D oocyte’s shape. Recombinant porcine ZP2 (C and N-terminus), ZP3 and ZP4 glycoproteins were expressed with peptide tags to allow their identification and conjugation to magnetic sepharose beads. Beads, with individual zona glycoproteins were studied: 1) for their ability to support adhesion of *in vitro* matured porcine cumulus cells; 2) their potential to bind spermatozoa; 3) their ability to induce acrosome exocytosis; and 4) determine if these interactions were affected by the protocol used for sperm capacitation. In summary, this system recreates a 3D environment of ovulated eggs that is scalable and will offer insights into molecular mechanisms of gamete recognition in mammals.

## Results

### Secreted recombinant ZP glycoproteins are stably and uniformly conjugated to beads

**Figure 1:**
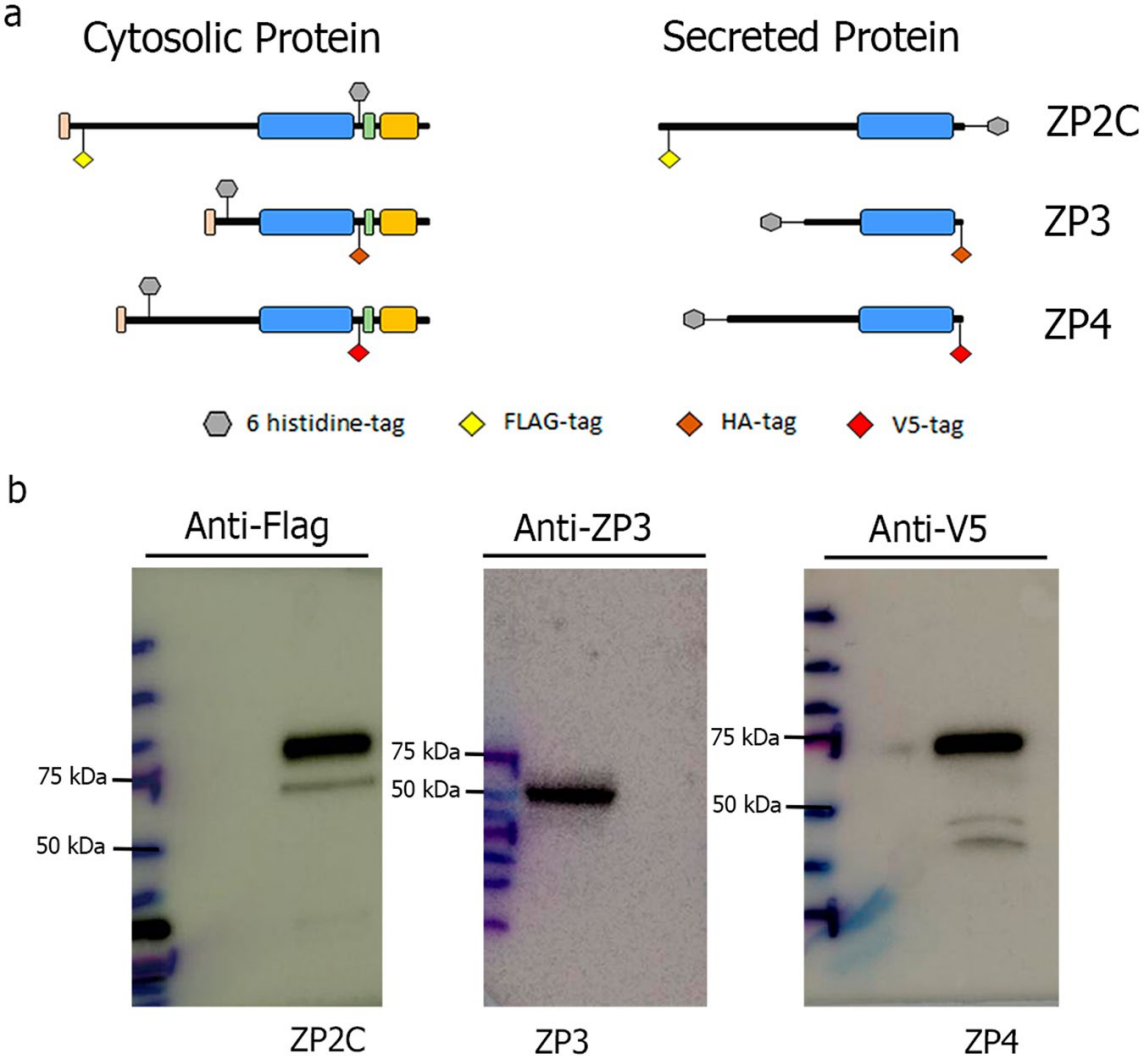
Design and expression of porcine recombinant ZP proteins. **(a**) Schematic representation of recombinant porcine ZP glycoproteins, ZP2C, ZP3 and ZP4. Signal peptide (pink), ZP domain (blue), processing region (green) and transmembrane domain (orange). (**b)** Proteins were expressed in CHO cells, separated by SDS-PAGE and analysed by western blot. ZP proteins were probed with anti-Flag antibodies for ZP2C, anti-ZP3 for ZP3 and V5 Epitope Tag antibody for ZP4. Molecular mass markers, left.

Expression plasmids encoding porcine ZP2C, ZP2N, ZP3 and ZP4 proteins (Fig. 1a, Supplementary Material Fig. S1) were expressed in Chinese Hamster Ovary (CHO) cells and secreted glycoproteins were successfully isolated. Each zona glycoprotein had the expected molecular mass^10^. ZP2C and ZP2N glycoproteins showed a molecular weight of 100 kDa, ZP3 reached 55 kDa, and ZP4 was 65 kDa on immunoblots probed with anti-Flag (ZP2C and ZP2N), anti-ZP3 (ZP3) and anti-V5 (ZP4) antibodies (Fig. 1b, Supplementary Material Fig. S1).

**Figure 2:**
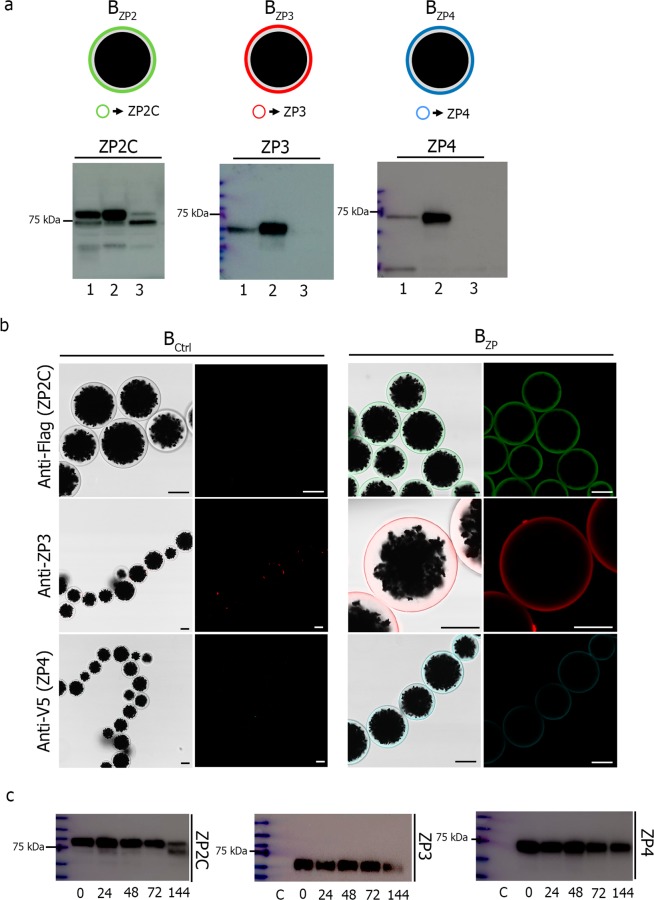
Conjugation of ZP recombinant proteins to sepharose magnetic beads. (**a**) Schematic representation of ZP recombinant proteins coated beads (B_ZP2,_ B_ZP3_ and B_ZP4_) (upper). SDS-PAGE and western blot of ZP2C, ZP3 and ZP4 proteins conjugated to magnetic beads (lower). Medium with secreted proteins before conjugation (lane 1), in the eluted fraction (lane 2), and media after conjugation (lane 3). Anti-Flag antibody was used for ZP2, anti-ZP3 for ZP3 and V5 Epitope Tag antibody for ZP4. (**b**) Confocal microscopy images of beads alone (B_Ctrl_) (left) and conjugated beads (B_ZP_) (right) showing uniform coating of beads with ZP proteins. Scale bar, 40 μm. (**c)** SDS-PAGE and western blot of ZP2C, ZP3 and ZP4 proteins conjugated to magnetic beads after conjugation and storage for 0, 24, 48, 72 and 144 h. Lane C, beads incubated with CHO-cell growth medium without recombinant zona proteins.

After incubation of beads with medium containing secretions from transfected CHO cells, all recombinant glycoproteins were successfully conjugated to beads (Fig. 2a). Electrophoresis and western blots confirmed their expected molecular weights (100 kDa for B_ZP2_, 55 kDa for B_ZP3_ and 65 kDa for B_ZP4_) both in the secreted medium before bead conjugation (lane 1) and after elution from the beads (lane 2). The proteins were not detected in the medium (lane 3) confirming that all the glycoprotein secreted in the CHO medium was successfully bound to beads. Similar findings were observed for B_ZP2N_ (Supplementary Material Fig. S1c) and eluted glycoproteins were analysed by mass spectrometry to confirm their identities. All the validated peptides from mass spectrometry had a Scored Peak Intensity (SPI) higher than 70% and a peptide score greater than 5 (Supplementary Material Fig. S2). Protein concentrations in the eluted medium were 0.157, 0.125 and 0.172 μg/μl for ZP2C, ZP3 and ZP4, respectively.

A thick and uniform coating of beads’ surface by recombinant ZP glycoproteins was confirmed by immunofluorescent confocal microscopy and no signal was observed in the beads incubated with CHO-cell medium alone (B_Ctrl_). Moreover, electrophoresis and western blots documented that adherence of recombinant glycoprotein to the sepharose beads was stable after storage for 0, 24, 48, 72 and 144 h (Fig. 2c and Supplementary Material Fig. S1c). Once both ZP2C and ZP2N were characterized subsequent assays used ZP2C because of greater abundance of ZP2C relatively to ZP2N which had an N-terminal histidine tag.

### ZP-coated beads form a 3D oocyte-like shape that supports sperm binding

**Figure 3:**
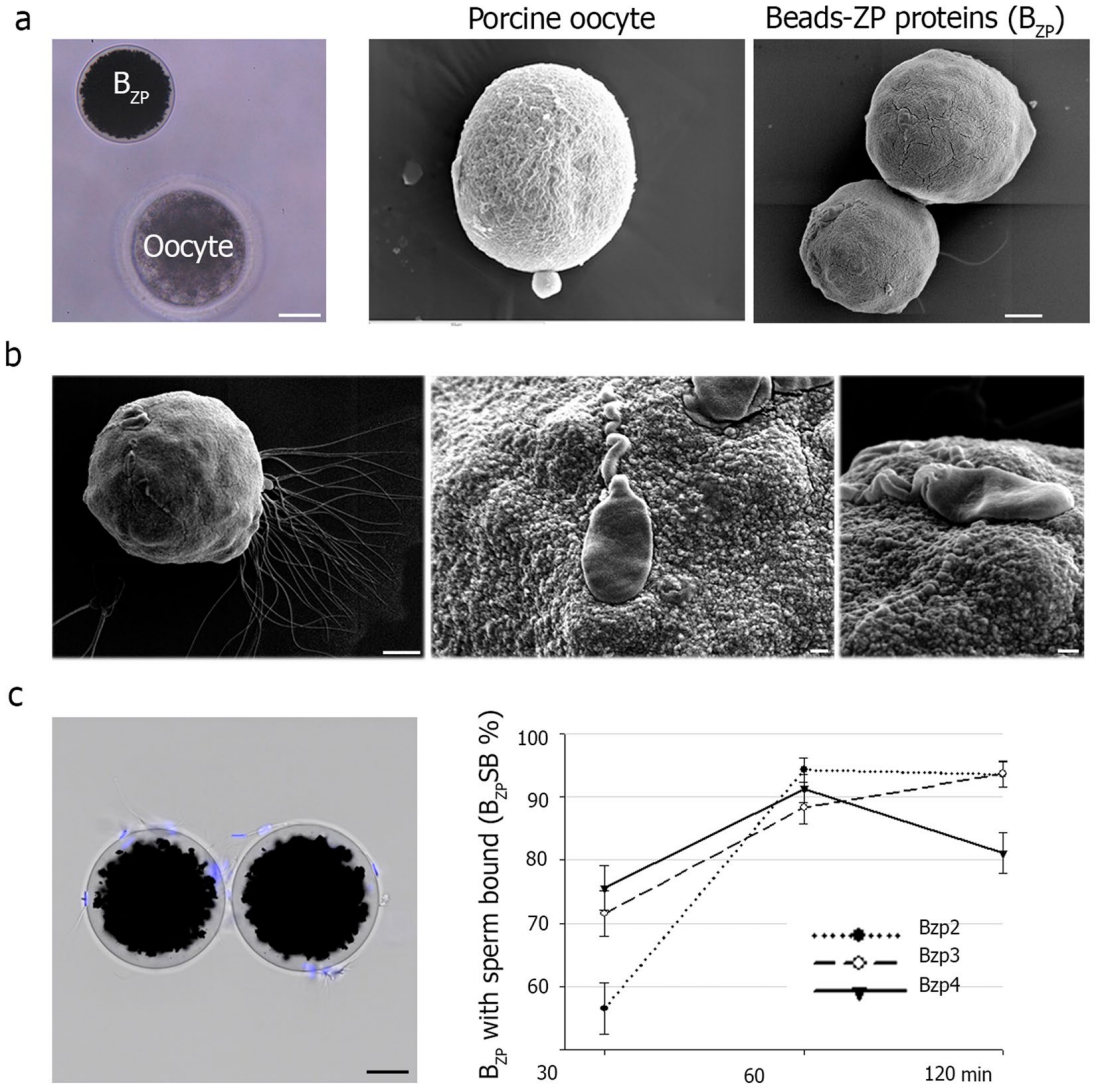
Sperm-B_ZP_ binding characterization. (**a**) Magnetic bead conjugated to ZP proteins (B_ZP_) and native denuded porcine oocyte observed by stereomicroscopy. Scale bar, 30 μm (left). Denuded porcine oocyte and beads conjugated with ZP proteins (B_ZP_) observed under scanning electron microscope. The typical sponge-like structure of the ZP is observed in the oocyte (middle) and is like the variegate surface of the recombinant zona protein-sepharose beads (right). Scale bar, 10 μm. (**b**) Scanning electron microscopy of ZP-beads (B_ZP_) after co-incubation with porcine spermatozoa. Scale bar, 10 μm (left). Higher magnification of sperm binding that documents small deformation mounds after interaction with the surface of a B_ZP_ (middle, right). Scale bar, 1 μm (**c**) ZP coated beads (B_ZP_) with attached porcine spermatozoa after co-incubation with 200.000 sperm cells/mL and observed under stereomicroscope (sperm DNA appear in blue after being stained with bisbenzimide; scale bar, 30 μm) (left). After co-incubation for 30, 60 and 120 min, B_ZP_ were fixed (0.5% glutaraldehyde), stained (0.01 mM bisbenzimide) and evaluated under epifluorescence microscope (right). The percentage of B_ZP_ with at least one sperm bound (B_ZP_SB) at each time was recorded.

Recombinant ZP glycoproteins conjugated to magnetic sepharose beads (B_ZP_) form 3D spheres (~120 μm) that approximates the size of native oocytes (Fig. 3a). Scanning electron microscopy provides images of a sphere with a rough surface that approximates the zona matrix of native oocytes (Fig. 3a). The spherical shape of B_ZP_ is an advantage over flat surfaces because it allows analysis of contact and binding of spermatozoa in three dimensions when live-imaged by stereo-, optic- and scanning-electron microscopy (Fig. 3b,c). In addition, the B_ZP_ transformed the kinetic energy of spermatozoa into deformation of the proteinaceous surface when they bind (Fig. 3b). Sperm-B_ZP_ binding assays were easily performed and recorded to evaluate sperm activity. The ability of ejaculated porcine sperm to bind to B_ZP2_, B_ZP3_ and B_ZP4_ varied among zona proteins over time (Fig. 3c). The number of B_ZP_ with at least one sperm bound (B_ZP_SB) increased with time and after 60 min, all preparations had more than 85% of the beads with at least one bound sperm.

### ZP-coated beads recapitulate the size and shape of native porcine cumulus-oocyte complexes (COC)

**Figure 4:**
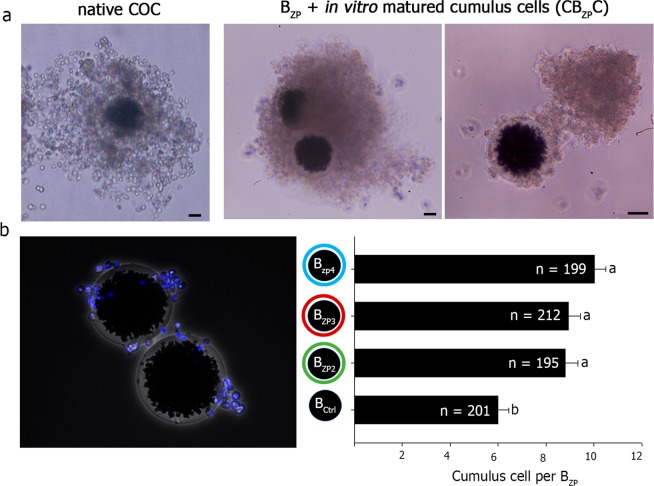
Mimicking the cumulus-oocyte complex (CB_ZP_C). (**a**) Expanded, native cumulus-oocyte complex (COC) after *in vitro* maturation. Scale bar, 30 μm (left). ZP-beads after 24 h of incubation with isolated cumulus cells obtained from *in vitro* matured COCs, cumulus-ZP coated beads complex (CB_ZP_C). Scale bar, 30 µm. The cells adhere to the bead surface resembling a native COC with two external coats, cumulus cells and ZP proteins (middle, right). (**b**) Quantification of the number of cells adhered to the ZP coated-bead (B_ZP_) stained with 0.01 mM bisbenzimide and mechanically washed (mean ± SEM). No difference in the number of adherent cells was observed among the three zona proteins attached to sepharose beads (B_ZP2,_ B_ZP3_, B_ZP4_). However, control beads without ZP proteins had fewer cells. Different letters (**a,b**) in the same column indicate differences between groups (P < 0.001).

After 24 h of co-incubation, *in vitro* matured cumulus cells adhered to the surface of B_ZP_. This recreated the size and shape of native porcine cumulus-oocyte complexes (COC) in which the cumulus cells and ZP glycoproteins form two layers external to the oocyte (Fig. 4a). The complex formed between cumulus cells and B_ZP_ (CB_ZP_C) persisted after been mechanically washed (Fig. 4b) and no differences in adherence was observed among the three recombinant zona glycoproteins. In each case, more than 95% of B_ZP_ had at least one cumulus cell attached after washing (data not shown). Interestingly, the control beads with no ZP proteins on their surface (beads incubated with growth CHO-cell medium; B_Ctrl_) showed a lower number of cumulus cells per bead (5.99 ± 0.45, n = 201) than B_ZP3_ (8.92 ± 0.52, n = 212), B_ZP4_ (10.01 ± 0.49, n = 199) (P < 0.001) and B_ZP2_ (8.79 ± 0.54, n = 195). No differences were found among the three recombinant zona glycoproteins (Fig. 4b).

### Acrosome integrity of sperm bound to ZP-coated beads

**Figure 5:**
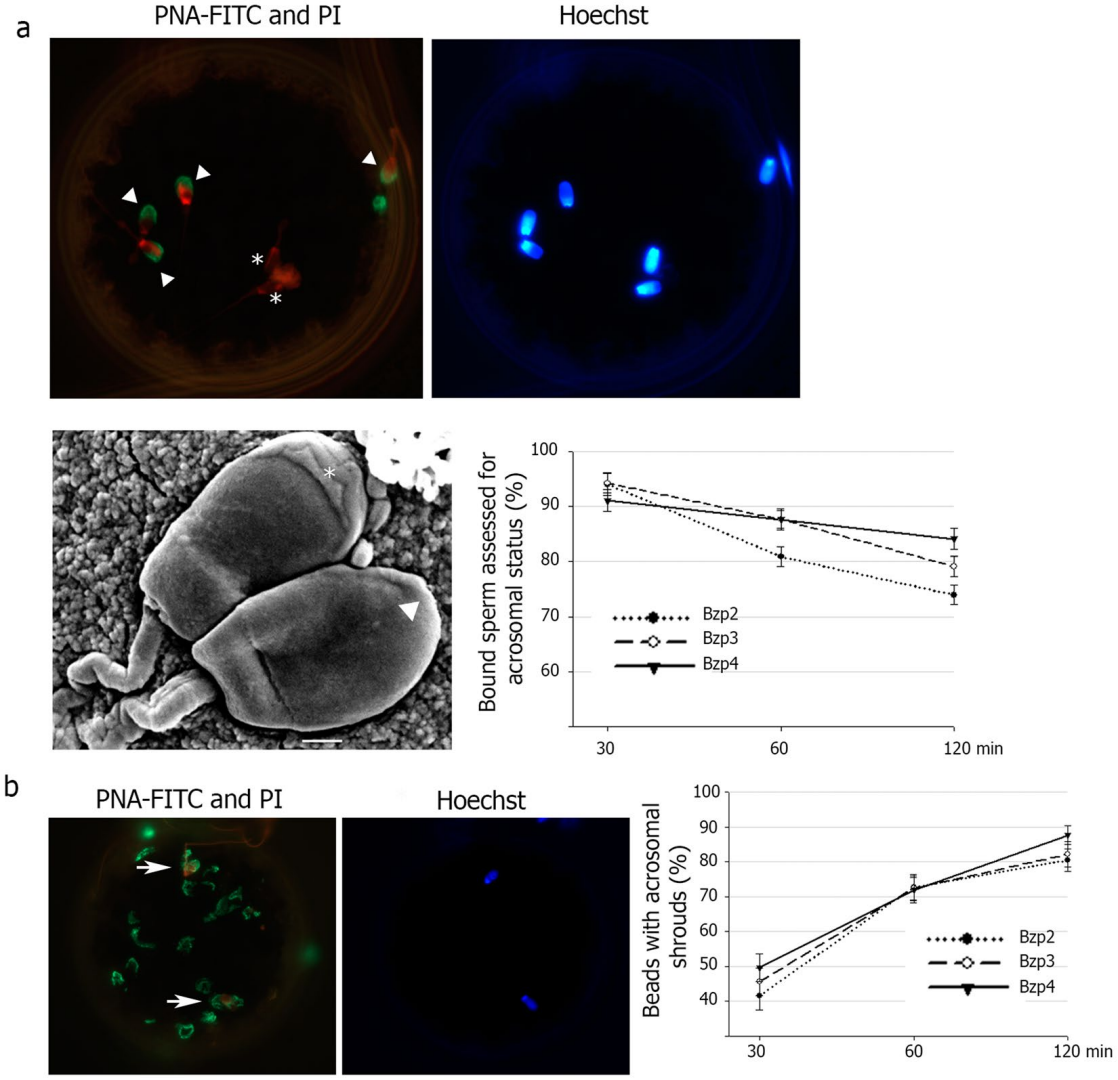
Acrosome status of sperm bound to the ZP-beads (B_ZP_). (**a**) B_ZP_ co-incubated with sperms were fixed (0.5% glutaraldehyde) and stained with 4 μg/mL PNA-FITC (fluorescein isothiocyanate-conjugated peanut agglutinin), 20 μg/mL PI (propidium iodide) and 0.01 mM bisbenzimide. Acrosome reacted (Δ) and non-reacted (*) spermatozoa were observed on the bead surface. Images taken from epifluorescence microscopy (upper) and scanning electron microscopy (lower left). Scale bar, 1 μm Percentage of sperm bound to B_ZP_ assessed for acrosome status over time of co-incubation (lower right). (**b**) Acrosomal shrouds on the surface of B_ZP_. Beads were fixed and stained with as in (**a**). Acrosomal shrouds (green) and spermatozoa heads (arrow) were observed by epifluorescence microscopy (left, middle). The number of B_ZP_ with acrosomal shrouds on the surface increased in a time-dependent manner (Ρ < 0.05) (right).

The acrosomal status of the sperm bound to beads was verified by means of PNA-FITC staining and was confirmed at the ultrastructural level by scanning electron microscopy (Fig. 5a). Acrosome status was recorded for 93% of bound sperm at 30 min, 85% at 60 min and 79% at 120 min (Fig. 5a). Exocytosis was observed on the surface of the three models (B_ZP2_, B_ZP3_, B_ZP4_) (Fig. 5b) and exocytosis increased with time. Forty-five per cent of B_ZP_ showed acrosomal shrouds on their surface after 30 min incubation, 70% of B_ZP_ at 60 min and over 80% of B_ZP_ at 120 min.

### Sperm binding to ZP-coated beads reflect method of capacitation

**Figure 6:**
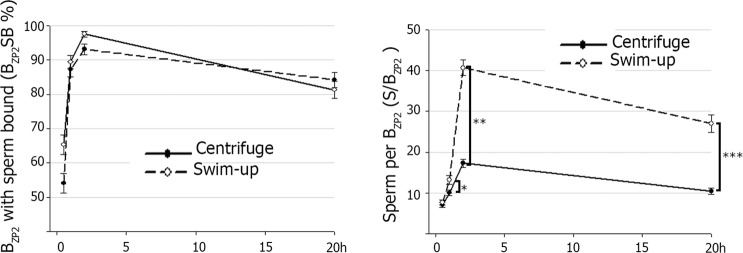
Sperm binding to ZP2C-coated beads (B_ZP2_) after capacitation by two different methods. B_ZP2C_ were incubated for 0.5, 1, 2 and 20 h with 200.000 porcine spermatozoa/mL capacitated by swim-up or double centrifugation method. After co-incubation, B_ZP2C_ were fixed (0.5% glutaraldehyde), stained (0.01 mM bisbenzimide) and evaluated by epifluorescence microscope to calculate the percent of B_ZP2_ with at least one sperm bound (B_ZP2_SB) (left) and to count the number of sperm bound per bead (S/B_ZP2_) (right). Higher number of sperm bound per B_ZP2_ after capacitation with double centrifugation was observed at 1 h (^*^Ρ = 0.015), 2 h (^**^Ρ < 0.001) and 20 h (***Ρ < 0.001).

B_ZP2C_ were incubated for 0.5, 1, 2 and 18 h with spermatozoa capacitated by either swim-up or double centrifugation and the number of beads with at least one sperm bound (B_ZP2_SB) and the number of sperm bound per bead (S/B_ZP2_) was determined (Fig. 6). With either method of capacitation, the B_ZP2_SB steadily increased for the first 2 h of co-incubation and then decreased after 18 h due to detachment of sperm from beads (Fig. 6 left panel). The temporal profile of S/B_ZP2_ was similar to B_ZP2_SB. No differences in the S/B_ZP2_ were found between sperm capacitation protocols at time 0.5 h, but a higher S/B_ZP2_ after capacitation with double centrifugation was observed at 1 h (13.21 ± 1.11 n = 245 *vs*. 10.04 ± 0.68 n = 253, Ρ = 0.015), 2 h (40.68 ± 1.93 n = 278 *vs*. 17.28 ± 0.98 n = 268, Ρ < 0.001) and 20 h (27.02 ± 2.14 n = 204 *vs*. 10.42 ± 0.74 n = 234, Ρ < 0.001) (Fig. 6 right panel).

## Discussion

The molecular basis of gamete recognition among mammals remains unclear. In gene-edited mice, Izumo^34^, Juno^35^, CD9^36^ and ZP2^20^ are required for sperm-egg recognition. However, these proteins have not been tested in other mammals, or the results have been controversial because of taxon-specific features of the fertilization. A significant impediment to obtaining unequivocal results has been the absence of a consistent assay for gamete recognition. Therefore, we have investigated zona glycoprotein- sepharose beads (B_ZP_) as a 3D model of mammalian gamete interactions that mimics the spherical shape of oocytes, easily translates among mammals and is scalable. Magnetic sepharose beads are conjugated to recombinant ZP glycoproteins to which cumulus cells and sperm bind and allow for assessment of acrosome status under varying experimental condition. This technology represents a necessary first step in validating the role of individual egg proteins in sperm-egg recognition. As an example, we now report testing three different zona glycoproteins potentially involved in porcine gamete interactions.

The zona proteins are processed to remove a signal peptide at their N-terminus and are released from the C-terminus transmembrane domain so that an ectodomain can participate in the extracellular zona pellucida that surrounds ovulated eggs^37–39^. Therefore, care needs to be taken in the placement of epitope tags to ensure that they are present in recombinant zona glycoproteins. Because of glycosylation and multiple disulfide bonds, it is preferred that heterologous mammalian cells lines be used for production to ensure secretion of biologically active recombinant zona glycoproteins even the glycosylation patterns differ from endogenous ZP proteins. Designed recombinant glycoproteins of the ZP were successfully secreted, detected by commercial antibodies and conjugated to magnetic sepharose beads to provide a homogeneous coating over the entire surface. The conjugation of recombinant zona glycoprotein to the beads was stable, allowing a wide window of time in which to perform desired experiments.

Inert B_ZP_ were selected because of their size, variegated surface and ease of handling. The native zona matrix porous matrix and its rough surface may be important for physiological responses when sperm bind to its surface^40^. Although individual recombinant ZP-proteins do not form native matrices, conjugated with sepharose beads provide a rough surface^41^ with sufficient stiffness that responds to mechanical pressure of sperm-ZP contact. According to current models, the kinetic energy of the sperm is converted into elastic deformation of the zona matrix necessary for successful penetration^42^. The sepharose-glycoprotein texture allows this deformation which mimics, to some extent, the mechanical properties of the ZP. Furthermore, two sperm-oocyte binding patterns have been described when observed by SEM and Transmission Electron Microscopy (TEM) techniques. The most commonly pattern, a flat and tangential attachment of the sperm head to the surface of the ZP, which is then followed by intrusion into the zona in precisely this horizontal position^43^ and a vertical sperm binding with ZP penetration by the tip of the head. This last pattern is found in oocytes where large, cluster-like numbers of bound spermatozoa are visible^44^. Both binding patterns are observed in the B_ZP_ models. Sperm bound to the bead surface showing apical binding is observed in Fig. 3b (left image) and a sperm flattened on bead surface is observed in Fig. 3b (centre and right images). Besides, some artefact during samples processing and also that sperm could be artificially forced to have additional interactions with the bead material when compared to the physiological configuration of the zona pellucida, are not discarded, as a slightly “rolled” way in the mid-pieces, head deformations and aphysiological mid-piece bead interaction that are observed in SEM pictures.

In most mammals, ovulated eggs are surrounded by a substantial layer of cumulus cells embedded in a hyaluronan matrix. The porcine cumulus cells-oocyte complex (COC) typically contains ~3000 cumulus cells^45^ and forms a barrier that sperm must penetrate as they approach the ZP. The spherical B_ZP_ and the characteristic of the binding surface of recombinant zona glycoproteins result in *de novo* cumulus cells adhesion. This recreates the cumulus matrix appearance of native COCs in which cumulus cells adopt teardrop-like shapes and projections penetrate the zona matrix^46^. The presence of recombinant zona glycoproteins increased the number of cumulus cells tightly attached to the surface of the B_ZP_. To the best of our knowledge, this is the first 3D model of gamete recognition that includes cumulus cells and provides a robust system to study the physiological activity of the fertilizing sperm.

To evaluate the role of individual zona proteins in gamete recognition, investigators have sought to assay sperm-binding activity and the ability to induce acrosome exocytosis. Two assays for sperm–ZP binding tests are the hemizona assay^47^ and a competitive intact zona sperm binding test^48^, but reported results have been contradictory^49^. Individual recombinant zona proteins seemingly provide simpler, more reproducible and physiologically relevant sperm–ZP binding and acrosome reaction assays^50^. Taking advantage of recombinant zona glycoproteins coupled to sepharose beads, we have validated a sperm-B_ZP_ binding assay using porcine gametes. Each B_ZP_ was treated as an individual oocyte using the same sperm:bead ratio commonly used in porcine IVF studies^8^ and had time-dependent binding as is observed with native gametes^51,52^. The acrosome status of bound spermatozoa was readily determined by staining with PNA-FITC (acrosome reacted) and confirmed by the time-dependent presence of acrosomal shrouds on surface of the B_ZP_^52,53^. While conducted using porcine gametes, the same design is easily translated to other species.

The method of sperm preparation affects gamete viability, motility, capacitation, ZP binding ability and acrosome exocytosis in both livestock^54–56^ and humans^57,58^. Swim-up selection is a more physiological approach than washing by centrifugation and sperm cells selected by this method have lower progressive motility, slower capacitation speed, lower ROS content and DNA fragmentation levels than those sperm selected by centrifugation^56,59,60^. Using B_ZP_, centrifuged sperm bound to the beads at a higher rate than swim-up sperm as is observed with native gametes^56^.

From a structural point of view, the B_ZP_ closely mimic native cumulus-oocyte complexes by maintaining a spherical shape, supporting cumulus cell adhesion and providing a glycoprotein-specific, variegated surface analogous to the native ZP. From a biological point of view, the B_ZP_ support sperm binding and recapitulates induction of acrosome exocytosis replete with the appearance of acrosomal shrouds and dependence of sperm binding on different methods of capacitation. From these observations, we conclude that the recombinant zona glycoprotein-coated beads will provide a valuable tool to explore the molecular basis of gamete recognition in a wide range of mammals. An additional movie file shows a graphical animation of the methodology developed (see Supplementary Movie).

## Methods

Unless otherwise stated, all chemicals were purchased from Sigma-Aldrich Química (Madrid, Spain). This study was carried out in strict accordance with the recommendations in the Guiding Principles for the Care and Use of Animals (DHEW Publication, NIH, 80–23). The protocol was approved by the Ethical Committee for Experimentation with Animals of the University of Murcia, Spain (Project Code: AGL2015-70159-P and AGL2015–66341‐R).

### Recombinant zona pellucida (ZP) glycoproteins design and expression

Expression plasmids (pcDNA3.1[+]) were designed and constructed to encode porcine ZP glycoproteins (ZP2: UniProt P42099; ZP3: UniProt P42098; and ZP4: UniProt Q07287) (GeneArt, Life Technology, Thermo Fisher Scientific). A histidine tag (6x) was added to the N-terminus of ZP2N (38–43 aa), ZP3 (41–46 aa) and ZP4 (22–27 aa), and to the C-terminus of ZP2C (644–649 aa). In addition, a FLAG-tag was added to ZP2C (38–45 aa) and ZP2N (642–649 aa); an HA tag was added to ZP3 (313–321 aa); and a V5 tag to ZP4 (469–482 aa). After verification by DNA sequence, ZP2C, ZP2N, ZP3 and ZP4 expression plasmids were amplified using Library Efficiency DH5α Competent cells (Thermo Fisher Scientific) and purified with GenEluted Plasmid Kit.

Chinese Hamster Ovary cells (CHO cells, ECACC, The European Collection of Authenticated Cell Cultures) were grown (37 °C, 5% CO_2_ and 95% humidity) for 48–72 h to 80–90% confluence using F-12 medium (Biowest, Nuaillé, France) supplemented with 10% fetal bovine serum (Biowest, Nuaillé, France) and 100 U/mL penicillin-streptomycin (GibcoBRL-Life Technologies, Gaithersburg, USA). Transient transfections were performed with X-tremeGene HP (Roche Applied Science, Indianapolis, USA) in accordance with the manufacturer’s protocol. For each transfection, 4 μL X-tremeGene HP transfection reagent were added to a final volume of 200 μL Opti-MEM reduced-serum medium (Gibco-Invitrogen) pre-dissolved with 2 μg template plasmid and incubated for 15 min at room temperature (RT). The complex was diluted by adding 2 ml Opti-MEM and overlaid on growing cells (37 °C, 5% CO_2_ and 95% humidity). The medium containing the secreted glycoproteins was collected after 48 h, centrifuged (3,939 × g, 5 min, 4 °C) to remove cell debris and concentrated in Vivaspin Turbo 4 of 10.000 Da (Sartorius), obtaining a final volume of 200–300 μL of concentrated glycoproteins in 20 mM sodium phosphate buffer, pH 7.4.

### Conjugation of recombinant glycoproteins to magnetic beads

Magnetic sepharose beads (His Mag Sepharose Excel GE Healthcare) were handled with a magnetic rack (MagRack 6, GE Healthcare). The magnetic sepharose beads were homogenously resuspended and 10 μL of bead slurry was pipetted into a microcentrifuge tube containing 500 μL of 20 mM sodium phosphate buffer, pH 7.4. The beads were washed with 500 μL of washing buffer (20 mM sodium phosphate, 0.5 M NaCl, 10 mM imidazole, pH 7.4) and finally with 500 μL of binding buffer (20 mM sodium phosphate, 0.5 M NaCl, 0.1% Tween-20, pH 7.4). Concentrated recombinant ZP glycoproteins (ZP2C, ZP2N, ZP3 and ZP4) were incubated with washed magnetic beads (1:1 v/v) overnight at 4 °C with orbital agitation. After incubation, the beads coated with glycoproteins (B_ZP2C_, B_ZP2N_, B_ZP3_ and B_ZP4_) were washed twice with 20 mM sodium phosphate buffer (pH 7.4) to remove non-conjugated proteins, resuspended in 20 mM sodium phosphate buffer and stored at 4 °C until use. As a control, beads were incubated with non-transfected CHO cells media processed as though it contained recombinant protein (B_Ctrl_). The recombinant zona glycoproteins were eluted by adding 100 μL of elution buffer (20 mM sodium phosphate, 0.5 M NaCl, 500 mM imidazole, pH 7.4) and incubated at 4 °C and 750 g in a thermomixer for 1 h. ZP2C, ZP3 and ZP4 glycoprotein concentrations were estimated by Bradford colorimetry at the Molecular Biology Service (SACE, University of Murcia, Spain). ZP2C, ZP3 and ZP4 were separated by SDS-PAGE and the bands corresponding to each glycoprotein were isolated and processed for proteomic analysis at the Molecular Biology Service (SACE, University of Murcia, Spain). The protein identity was determined by mass spectrometry, carried out by using a HPLC/MS system, in which an Agilent 1100 Series HPLC (Agilent Technologies, Santa Clara, CA, USA) is connected to an Agilent Ion Trap XCT Plus Mass Spectrometer (Agilent Technologies, Santa Clara, CA, USA) using an electrospray (ESI). Data processing was performed by the Data Analysis program for LC/MSD Trap Version 3.3 (Bruker Daltonik, GmbH, Germany) and Spectrum Mill MS Proteomics Workbench (Rev A.03.02.060B, Agilent Technologies, Santa Clara, California, USA).

### Western blot

Cell growing medium containing concentrated glycoproteins was separated by SDS-PAGE and transferred to PVDF membranes which were probed with antibodies: anti-Flag for ZP2 (1:1000 v/v in TBST 1x, 1% BSA); anti-ZP3 kindly donated by Dr. Hedrick^61^, for ZP3 (1:2000 v/v in TBST 1x, 1% BSA); and V5 Epitope Tag Antibody for ZP4 (1:2000 v/v in TBST 1x, 1% BSA)(Thermo Fisher Scientific) prior to visualization by chemiluminescence (Pierce ECL-Plus, Thermo Fisher Scientific).

B_ZP_ were analyzed by SDS-PAGE to verify that recombinant glycoproteins were successfully bound to the sepharose beads. To study the stability of the conjugation, after storage (4 °C, 20 mM sodium phosphate buffer, pH 7.4) for 0, 24, 48, 72 and 144 h, the B_ZP_ were washed twice with 20 mM sodium phosphate buffer, pH 7.4, resuspended in 20 mM sodium phosphate buffer, pH 7.4 and solubilized under reducing conditions in 4X SDS sample buffer (Millipore, USA). After 10 min at 100 °C, the supernatant was separated by SDS-PAGE, transferred to PVDF membranes and probed with the aforementioned ZP antibodies.

### Confocal microscopy

After conjugation, the B_ZP_ were incubated (1 h, RT) with antibodies diluted in calcium- and magnesium-free PBS supplemented with 1% BSA. Antibody dilutions were: anti-FLAG for ZP2 (1:200 v/v); anti-ZP3 for ZP3 (1:200 v/v); and V5 Epitope Tag Antibody for ZP4 (1:200 v/v). The B_ZP_ then were incubated (1 h, RT) with secondary antibodies (anti-rabbit IgG-FITC conjugate (1:100 v/v) for ZP2 and ZP4; Alexa Fluor 488 Donkey anti-goat IgG (1:200 v/v) for ZP3), washed in PBS and fixed with 2% paraformaldehyde (Electron Microscopy Sciences, Hatfield, Philadelphia, USA). Stained B_ZP_ were placed on a chambered slide (25-μL cavity) with Gene Frame (Advanced Biotechnologies, Leatherhead, UK) and covered with a coverslip. Samples were analysed with a Leica TCS SP8 confocal microscope and image analysis was performed using LAS X Core software analysis software (Leica Microsystems, Spain). FITC and Alexa Fluor 488 were excited at 488 nm.

### Incubation of ZP-conjugated beads with isolated *in vitro* matured cumulus cells

To recreate an *in vitro* 3D model resembling a cumulus-oocyte complex, the models B_ZP2C_, B_ZP3_ and B_ZP4_ were incubated with cumulus cells derived from *in vitro* matured porcine cumulus oocyte complexes (COCs)^52^. COCs were isolated from ovaries obtained from 6 to 7-month-old animals slaughtered at an abattoir. After *in vitro* maturation, COCs were decumulated in PBS-1% BSA by pipetting, oocytes were discarded, and cumulus cells were centrifuged twice (1,200× g, 10 min) in modified TALP medium^62^. B_ZP_ (50–55) were incubated (24 h, 38.5 °C, 5% CO_2_, 20% O_2_ and saturated humidity) with 2,500 isolated cumulus cells/ B_ZP_^63^ in 500 μl TALP Adhesion of cumulus cells was confirmed microscopically by observing formation of cumulus mass plugs around coated beads (CB_ZP_C). CB_ZP_C were then gently pipetted three times in PBS supplemented with 0.1% PVA to remove loosely adherent cumulus cells, fixed with 0.5% (v/v) glutaraldehyde, stained with 0.01 mM Hoechst 33342, mounted on cover slips and observed by epifluorescence microscope at 40× . Three double-blind replicates were obtained to determine the number of cumulus cells remaining attached to the bead surface.

### Sperm preparation

Ejaculated spermatozoa from boars proven to be fertile (Landrace × Largewhite, 12–24 months old; CEFU, SA, Pliego, Murcia, Spain) from Artificial Insemination Center (AIC) were diluted (1:5 v/v) in Beltsville Thawing Solution (BTS). To validate sperm binding to the B_ZP_, heterospermic samples were prepared either by double centrifugation^64^ or swim-up^56^. For the double centrifugation, diluted sperm samples were centrifuged (300× g, 3 min) after which the supernatant was collected and centrifuged again (800 × g, 5 min). The supernatant was discarded, and the sperm pellet was resuspended in modified TALP medium^62^ supplemented with 1 mM sodium pyruvate, 0.6% BSA and 50 μg/mL gentamycin. For the swim-up protocol, 1 ml diluted semen sample was laid below 1 ml NaturARTs PIG sperm swim-up medium (EmbryoCloud, Murcia, Spain) at the bottom of a conical tube. After 20 min at 37 °C (with the tube at a 45° angle), 800 μl from the top of the tube were aspirated. Final sperm concentrations were adjusted to 200,000 sperm cells/mL with supplemented modified TALP media.

### Scanning electron microscopy

B_ZP_ co-incubated (2 h) with double centrifuged sperm were fixed (1% glutaraldehyde in PBS (v/v), 30 min), washed three times in molecular biology water and transferred to dry on a coverslip. Samples were then processed by the General Unit of Microscopy (University of Alicante, Spain). Finally, the beads were observed under an electron microscope JEOL JSM 840 SEM (Jeol Limited, London, United Kingdom). The shape and surface characteristics of the B_ZP_ as well as the acrosome integrity of the bound sperm were recorded.

### Sperm-BZP binding assay

B_ZP_ (50–55) conjugated with ZP2C, ZP3, ZP4 were washed twice in TALP medium and co-incubated (38.5 °C, 5% CO_2_, 20% O_2_ and saturated humidity) with double centrifuged spermatozoa (200,000 sperm/mL) in 500 μl medium. In addition, B_ZP2C_ were inseminated with sperm prepared by swim-up (200,000 sperm/mL) to validate B_ZP2C_ as a suitable model for sperm-ZP recognition independent of the method of sperm preparation. To assess the number of sperm bound and the integrity of their acrosomes after 0.5, 1, 2, and 20 h of coincubation, an aliquot of B_ZP2C_ was washed three times in PBS, supplemented with 0.1% PVA, fixed with 0.5% glutaraldehyde in PBS (v/v), and stained with 0.01 mM Hoechst 33342, 4 μg/mL fluorescein isothiocyanate-conjugated peanut agglutinin (PNA-FITC) and 20 μg/mL of propidium iodide (PI). B_ZP_ were mounted on slides, and the number of sperms bound and acrosome status (acrosome from reacted sperm stains green), were recorded under an epifluorescence microscopy (Leica, DMLS, Barcelona, Spain). The percent of beads with at least one sperm bound (B_ZP_SB) and the mean number of sperm per bead (S/B_ZP_) were recorded and acrosome integrity was evaluated. Spermatozoa with damaged acrosome and acrosomal content were observed green. Three replicates were performed.

### Statistical analysis

Data are presented as mean ± SEM and all percentages were modelled according to the binomial model of variables and arcsine transformation to achieve normal distribution. The percent of beads with at least one sperm bound, number of sperm per bead, beads with at least one cumulus cell attached and the mean number of cumulus cells per bead were analysed by one-way ANOVA. When ANOVA revealed a significant effect, values were compared by Tukey’s test. A Ρ value < 0.05 was considered statistically significant. The analysis was conducted using Systat v13.1 (Systat Software, Inc San Jose, CA, USA).

## Supplementary information

Supplementary info

Supplemetary Movie

## References

[1] Bronson RA, McLaren A (1970). Transfer to mouse oviduct of eggs with and without zona pellucida. J Reprod Fertil..

[2] Rankin T (1996). Mice homozygous for an insertional mutation in the Zp3 gene lack a zona pellucida and are infertile. Development.

[3] Rankin TL (2001). Defective zonae pellucidae in Zp2-null mice disrupt folliculogenesis, fertility and development. Development.

[4] Gupta SK (2012). Mammalian zona pellucida glycoproteins: structure and function during fertilization. Cell and Tissue Res..

[5] Avella MA, Xiong B, Dean J (2013). The molecular basis of gamete recognition in mice and humans. Mol Hum Reprod..

[6] Brito IR (2014). Three-dimensional systems for *in vitro* follicular culture: overview of alginate-based matrices. Reprod Fertil Dev..

[7] Ferraz M, Henning HHW, Stout TAE, Vos P, Gadella BM (2017). Designing 3-Dimensional *In Vitro* Oviduct Culture Systems to Study Mammalian Fertilization and Embryo Production. Ann Biomed Eng..

[8] Romar R, Funahashi H, Coy P (2016). *In vitro* fertilization in pigs: New molecules and protocols to consider in the forthcoming years. Theriogenology..

[9] Goudet G, Mugnier S, Callebaut I, Monget P (2008). Phylogenetic analysis and identification of pseudogenes reveal a progressive loss of zona pellucida genes during evolution of vertebrates. Biol Reprod..

[10] Hedrick JL, Wardrip NJ (1987). On the macromolecular composition of the zona pellucida from porcine oocytes. Dev Biol..

[11] Noguchi S (1994). Characterization of the zona pellucida glycoproteins from bovine ovarian and fertilized-eggs. Biochim Biophys Acta..

[12] Hoodbhoy T (2005). Human sperm do not bind to rat zonae pellucidae despite the presence of four homologous glycoproteins. J Biol Chem..

[13] Izquierdo-Rico MJ (2009). Hamster zona pellucida is formed by four glycoproteins: ZP1, ZP2, ZP3, and ZP4. J Proteome Res..

[14] Ganguly A, Sharma RK, Gupta SK (2008). Bonnet monkey (Macaca radiata) ovaries, like human oocytes, express four zona pellucida glycoproteins. Mol Reprod Dev..

[15] Lefievre L (2003). Proteomic analysis of five oocytes demonstrates that the human zona pellucida is composed of four major glycoproteins. Hum Reprod..

[16] Bleil JD, Wassarman PM (1980). Mammalian sperm-egg interaction: identification of a glycoprotein in mouse egg zonae pellucidae possessing receptor activity for sperm. Cell.

[17] Ganguly A (2010). In humans, zona pellucida glycoprotein-I binds to spermatozoa and induces acrosomal exocytosis. Hum Reprod..

[18] Yonezawa N, Kanai-Kitayama S, Kitayama T, Hamano A, Nakano M (2012). Porcine zona pellucida glycoprotein ZP4 is responsible for the sperm-binding activity of the ZP3/ZP4 complex. Zygote..

[19] Suzuki K (2015). The Hinge Region of Bovine Zona Pellucida Glycoprotein ZP3 Is Involved in the Formation of the Sperm-Binding Active ZP3/ZP4 Complex. Biomolecules..

[20] Avella MA, Baibakov B, Dean J (2014). A single domain of the ZP2 zona pellucida protein mediates gamete recognition in mice and humans. J Cell Biol..

[21] Baibakov B, Boggs NA, Yauger B, Baibakov G, Dean J (2012). Human sperm bind to the N-terminal domain of ZP2 in humanized zonae pellucidae in transgenic mice. Journal of Cell Biol..

[22] Bhandari B, Bansal P, Talwar P, Gupta SK (2010). Delineation of downstream signalling components during acrosome reaction mediated by heat solubilized human zona pellucida. Reprod Biol Endocrin..

[23] Franken DR, Henkel R, Kaskar K, Habenicht UF (1996). Defining bioassay conditions to evaluate sperm zona interaction: Inhibition of zona binding mediated by solubilized human zona pellucida. *J Assist Reprod*. Gen..

[24] Tesarik J, Mendoza C, Ramirez JP, Moos J (1993). Solubilized human zona pellucida competes with a fucosylated neoglycoprotein for binding sites on the human sperm surface. Fertil Steril..

[25] Chiu PCN (2008). Native human zona pellucida glycoproteins: purification and binding properties. Hum Reprod..

[26] Caballero-Campo P (2006). Biological effects of recombinant human zona pellucida proteins on sperm function. Biol Reprod..

[27] Vanduin M (1994). Recombinant human zona pellucida protein ZP3 produced by chinese hamster ovary cells induces the human sperm acrosome reaction and promotes sper-egg fusion. Biol Reprod..

[28] Yonezawa N (2005). Recombinant porcine zona pellucida glycoproteins expressed in Sf9 cells bind to bovine sperm but not to porcine sperm. J Biol Chem..

[29] Aitken RJ, Richardson DW (1981). Measurement of the sperm binding capacity of the mouse zona pellucida and its use in the estimation of anti-zona antibody titres. J Reprod Fertil..

[30] Avella Matteo A., Baibakov Boris A., Jimenez-Movilla Maria, Sadusky Anna Burkart, Dean Jurrien (2016). ZP2 peptide beads select human sperm in vitro, decoy mouse sperm in vivo, and provide reversible contraception. Science Translational Medicine.

[31] Okabe M (2013). The cell biology of mammalian fertilization. Development..

[32] Chiu PCN, Lam KKW, Wong RCW, Yeung WSB (2014). The identity of zona pellucida receptor on spermatozoa: An unresolved issue in developmental biology. Semin Cell Dev Biol..

[33] Wright GJ, Bianchi E (2016). The challenges involved in elucidating the molecular basis of sperm-egg recognition in mammals and approaches to overcome them. Cell Tissue Res..

[34] Inoue N, Ikawa M, Isotani A, Okabe M (2005). The immunoglobulin superfamily protein Izumo is required for sperm to fuse with eggs. Nature..

[35] Bianchi E, Doe B, Goulding D, Wright GJ (2014). Juno is the egg Izumo receptor and is essential for mammalian fertilization. Nature..

[36] Miyado K (2000). Requirement of CD9 on the egg plasma membrane for fertilization. Science..

[37] Boja ES, Hoodbhoy T, Fales HM, Dean J (2003). Structural characterization of native mouse zona pellucida proteins using mass spectrometry. J Biol Chem..

[38] Jovine L, Qi HY, Williams Z, Litscher ES, Wassarman PM (2004). A duplicated motif controls assembly of zona pellucida domain proteins. Proc. Natl. Acad. Sci. USA.

[39] Jimenez-Movilla M, Dean J (2011). ZP2 and ZP3 cytoplasmic tails prevent premature interactions and ensure incorporation into the zona pellucida. J Cell Sci..

[40] Baibakov B, Gauthier L, Talbot P, Rankin TL, Dean J (2007). Sperm binding to the zona pellucida is not sufficient to induce acrosome exocytosis. Development..

[41] Ghaeidamini M, Kharat AN, Haertle T, Ahmad F, Saboury A (2018). A. beta-Cyclodextrin-Modified Magnetic Nanoparticles Immobilized on Sepharose Surface Provide an Effective Matrix for Protein Refolding. J Phys Chem. A. B.

[42] Gefen A (2010). The Relationship Between Sperm Velocity and Pressures Applied to the Zona Pellucida During Early Sperm-Oocyte Penetration. J Bopmech Eng-T Asme..

[43] Sathananthan AH, Trounson AO, Wood C, Leeton JF (1982). Ultrastructural observations on the penetration of human-sperm into the zona pellucida of the human egg *in vitro*. J Androl..

[44] Familiari G, Heyn R, Relucenti M, Sathananthan H (2008). Structural changes of the zona pellucida during fertilization and embryo development. Front Biosci-Landmrk..

[45] Okudaira Y, Wakai T, Funahashi H (2017). Levels of cyclic-AMP and cyclic-GMP in porcine oocyte-cumulus complexes and cumulus-free oocytes derived from small and middle follicles during the first 24-hour period of *in vitro* maturation. J Reprod Develop..

[46] Tanghe S, Van Soom A, Nauwynck H, Coryn M, De Kruif A (2002). Minireview: Functions of the cumulus oophorus during oocyte maturation, ovulation, and fertilization. Mol Reprod Dev..

[47] Burkman LJ (1988). The hemizona assay (HZA): development of a diagnostic test for the binding of human spermatozoa to the human hemizona pellucida to predict fertilization potential. Fertil Steril..

[48] Liu DY, Lopata A, Johnston WIH, Baker HWG (1988). A human sperm-zona pellucida binding test using oocytes that failed to fertilize *in vitro*. Fertil Steril..

[49] Waberski D (2005). Importance of sperm-binding assays for fertility prognosis of porcine spermatozoa. Theriogenology.

[50] Franken DR, Bastiaan S, Oehninger SC (2000). Physiological induction of the acrosome reaction in human sperm: Validation of a microassay using minimal volumes of solubilized, homologous zona pellucida. J Assist Reprod Genet..

[51] Coy P (1993). *In vitro* fertilization of pig oocytes after different coincubation intervals. Theriogenology..

[52] Romar R, Coy P, Rath D (2012). Maturation conditions and boar affect timing of cortical reaction in porcine oocytes. Theriogenology..

[53] Katayama M, Koshida M, Miyake M (2002). Fate of the acrosome in ooplasm in pigs after IVF and ICSI. Hum Reprod..

[54] Matas C (2003). Effect of sperm preparation method on *in vitro* fertilization in pigs. Reproduction..

[55] Abraham MC, Johannisson A, Morrell JM (2016). Effect of sperm preparation on development of bovine blastocyst *in vitro*. Zygote..

[56] Canovas S (2017). DNA methylation and gene expression changes derived from assisted reproductive technologies can be decreased by reproductive fluids. Elife.

[57] Thijssen A (2014). Influence of temperature and sperm preparation on the quality of spermatozoa. Reprod Biomed Online..

[58] Kim SW, Jee BC, Kim SK, Kim SH, Sperm DNA (2017). fragmentation and sex chromosome aneuploidy after swim-up versus density gradient centrifugation. Clin Exp Reprod Med..

[59] Menkveld R, Swanson RJ, Kotze TJV, Kruger TF (1990). Comparison of a discontinuous Percoll gradient method versus a swim-up method: effects on sperm morphology and other semen parameters. Andrologia.

[60] Zini A, Finelli A, Phang D, Jarvi K (2000). Influence of semen processing technique on human sperm DNA integrity. Urology..

[61] Berger T, Davis A, Wardrip NJ, Hedrick JL (1989). Sperm binding to the pig zona pellucida and inhibition of binding by solubilized components of the zona pellucida. J Reprod Fertil..

[62] Rath D (1999). *In vitro* production of sexed embryos for gender preselection: High-speed sorting of X-chromosome-bearing sperm to produce pigs after embryo transfer. J Anim Sci..

[63] Campos I, Coy P, Romar R, Ruiz S, Gadea J (2001). Effects of maturational stage, cumulus cells and coincubation of mature and immature cumulus-oocyte complexes on *in vitro* penetrability of porcine oocytes. Theriogenology..

[64] Matas C (2010). Sperm treatment affects capacitation parameters and penetration ability of ejaculated and epididymal boar spermatozoa. Theriogenology..

