# Supplementary material for: Mammalian spermatozoa and cumulus cells bind to a 3D model generated by recombinant zona pellucida protein-coated beads
Source: Sci Rep. 2019 Nov 29;9:17989. doi: 10.1038/s41598-019-54501-7 (PMC6884566; doi:10.1038/s41598-019-54501-7)
Supplement: Supplementary file 1 — Supplementary info [file 41598_2019_54501_MOESM1_ESM.pdf]

Supplementary Material

Mammalian spermatozoa and cumulus cells bind to a 3D model generated by recombinant zona pellucida protein-coated beads.

Julieta Gabriela Hamze[1], Analuce Canha-Gouveia[2], Blanca Algarra[1], María José Gómez-Torres[3], María Concepción Olivares[4], Raquel Romar[2]* and María Jiménez-Movilla[1]*.

[1]Department of Cell Biology and Histology, School of Medicine, University of Murcia, Campus Mare Nostrum and IMIB-Arrixaca, Murcia, Spain. [2]Department of Physiology, Faculty of Veterinary, University of Murcia, Campus Mare Nostrum and IMIB-Arrixaca, Murcia, Spain. [3]Department of Biotechnology, Chair Human Fertility, University of Alicante, Spain. [4]Department of Biochemistry and Molecular Biology B and Immunology School of Medicine, University of Murcia, Campus Mare Nostrum and IMIB-Arrixaca, Murcia, Spain

Julieta Gabriela Hamze (julietagabriela.hamze@um.es), Analuce Canha-Gouveia (analuce.canha@um.es), Blanca Algarra (b.algarraonate@um.es), María José Gómez-Torres (mjose.gomez@ua.es), María Concepción Olivares (mcolisan@um.es), Raquel Romar (rromar@um.es), María Jimenez-Movilla (mariajm@um.es).

*Correspondence: mariajm@um.es, rromar@um.es

[1]Department of Cell Biology and Histology, School of Medicine, Instituto Murciano de Investigación Biosanitaria (IMIB-Arrixaca-UMU), University of Murcia, Campus Mare Nostrum, Murcia, Spain, 30100.

Supplementary Material Fig. S1

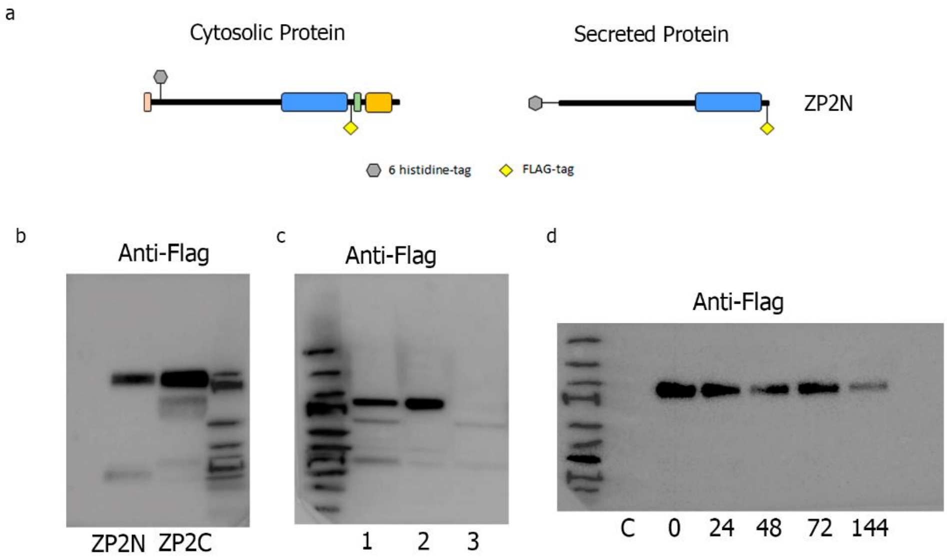

Supplementary Material Fig. S1. Design and expression of porcine recombinant ZP2N protein.

a Schematic representation of recombinant porcine ZP2N glycoprotein. Signal peptide (pink), ZP domain (blue), processing region (green) and transmembrane domain (orange). **b** Proteins were expressed in CHO cells, separated by SDS-PAGE and analyzed by western blot. ZP2N protein was probed with anti-Flag antibody. **c** Media with secreted proteins before conjugation (lane 1), in the eluted fraction (lane 2) and media after conjugation time (lane 3). **d** SDS-PAGE and western blot of ZP2N protein after conjugation to sepharose magnetic beads and storage for 0, 24, 48, 72 and 144 h. Lane C, beads incubated with CHO-cell growth medium without recombinant zona proteins. Molecular mass markers, left.

Supplementary Material Fig S2.
Table S1. Details of the peptides identified and validated from the trypsin digest by MS.

Protein Name	Score	SPI%	Sequence
Zona pellucida sperm-binding protein 2	17.99	89.0	(K)VHSHQTKPALNLDTLR(V)
Zona pellucida sperm-binding protein 2	14.95	89.7	(K)VIYENEIHALWADPPSAVSR(D)
Zona pellucida sperm-binding protein 2	13.42	83.7	(K)APAQGLVQFR(I)
Zona pellucida sperm-binding protein 2	15.95	97.9	(K)SVNLGSGNIAVSQLHK(H)
Zona pellucida sperm-binding protein 2	9.19	96.8	(R)MVVEFPR(I)
Zona pellucida sperm-binding protein 2	8.99	83.1	(R)QPIYLEVR(I)
Zona pellucida sperm-binding protein 2	8.22	98.1	(R)LIDDNAALR(Q)
Zona pellucida sperm-binding protein 2	7.70	95.4	(R)qEALmYHISCPVmGAEGPDQHSGSTIcmKDFmSFTFNFFPGmADENVKR(E)
Zona pellucida sperm-binding protein 2	5.30	73.5	(R)VGDSScQPTFK(A)

Protein Name	Score	SPI%	Sequence
Zona pellucida sperm-binding protein 3	14.44	88.3	(R)AEVPIEcHYPR(Q)
Zona pellucida sperm-binding protein 3	14.17	92.9	(R)WSPVEGPAVIcR(C)
Zona pellucida sperm-binding protein 3	11.34	79.5	(K)cEPLVSQDTDAVVR(F)
Zona pellucida sperm-binding protein 3	8.02	71.5	(R)NTIYITcHLK(V)
Zona pellucida sperm-binding protein 3	6.53	71.0	(K)MTPTFQLGDR(A)
Zona pellucida sperm-binding protein 3	5.44	70.2	(K)ACsFSKsSNR(W)
Zona pellucida sperm-binding protein 3	5.19	84.3	(R)LMEENWSAEK(M)

Protein Name	Score	SPI%	Sequence
Zona pellucida sperm-binding protein 4	17.55	91.5	(R)LPcAPPPITQGEcK(Q)
Zona pellucida sperm-binding protein 4	15.21	91.3	(R)VTGNQAVYENELVAAR(D)
Zona pellucida sperm-binding protein 4	9.55	94.2	(K)ASNLLFPSHYQR(F)
Zona pellucida sperm-binding protein 4	7.99	92.9	(R)FPFSScGTAK(R)
Zona pellucida sperm-binding protein 4	5.09	88.3	(R)EPIYVEVSIR(H)

Supplementary Material Fig. S2. Mass spectrometry of recombinant zona proteins.

Details of the peptides identified and validated from the trypsin digestion and analysis by mass spectrometry of porcine recombinant zona proteins.

Supplementary Material Fig. S3

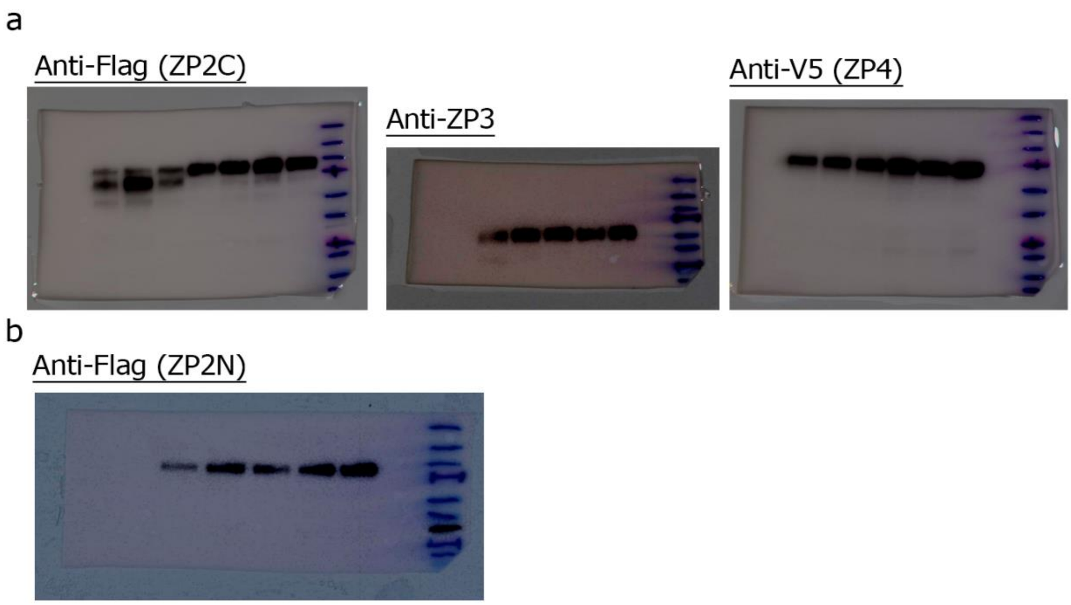

Supplementary Material Fig. S3. Full-length blots. a Full-length blots from figure 2c. **b** Full-length blot form supplementary material Fig. S1d.
